# **A qualitative study of the mental health outcomes in people being treated for obesity and type 2 diabetes with glucagon-like peptide-1 receptor agonists**

**DOI:** 10.1007/s00592-024-02392-0

**Published:** 2024-11-09

**Authors:** Aureliane C. S. Pierret, Madeleine Benton, Piya Sen Gupta, Khalida Ismail

**Affiliations:** 1 Department of Diabetes, St Thomas’ Hospital, Guy’s and St Thomas’ NHS Foundation Trust, Westminster Bridge Road, London, SE1 7EH UK; 2 Department of Psychological Medicine, King’s College London, 16 De Crespigny Park, London, SE5 8AB UK

**Keywords:** Obesity, Diabetes, GLP-1 receptor agonist, Depression, Binge-eating disorder, Qualitative

## Abstract

**Objective:**

Obesity and type 2 diabetes (T2D) are associated with increased rates of mental disorders, particularly depression, anxiety and binge-eating disorder. GLP-1 receptor agonists are a novel class of pharmacological agents for obesity and T2D. We aimed to describe participants’ experiences of GLP-1 receptor agonists on their mental health.

**Methods:**

Qualitative, individual, semi-structured interviews were conducted in nine participants who were prescribed GLP-1 receptor agonists for the treatment of obesity and/or T2D. Mental health status was measured at time of GLP-1 receptor agonist initiation and assessed again at 12–16 weeks when the semi-structured interview took place. Data were analysed using reflexive thematic analysis.

**Results:**

Three main themes were generated from the analysis: (1) acceptance of negative side effects for long term physical health benefits; (2) reflections on the diverse impact on mental health; (3) reduced appetite and increased control of eating behaviours.

**Discussion:**

Overall, participants with obesity and/or T2D described a positive impact of GLP-1 receptor agonists on their mental health, especially perception of improved control of eating behaviours. This suggests GLP-1 receptor agonists should be further studied for their potential effectiveness for treatment of binge-eating disorder.

**Supplementary Information:**

The online version contains supplementary material available at 10.1007/s00592-024-02392-0.

## Introduction

Glucagon-like peptide-1 (GLP-1) receptor agonists are a class of drugs developed initially for treatment of type 2 diabetes (T2D) and then obesity, by mimicking the incretin effect which includes glucose-dependent increased insulin secretion and improved glycaemic control, and inducing weight loss in a dose-dependent manner [1]. Obesity and T2D are both major global health challenges [2] with obesity being the main modifiable risk factor for T2D [3]. People with both obesity and T2D have a 7-fold increased risk of mortality compared to those with neither condition [4].

Obesity and T2D are strongly associated with mental disorders. Obesity has a bidirectional relationship with depression [5] and is associated with higher rates of anxiety [6]. Individuals with T2D are also at increased risk of depression [7] and anxiety [8]; this comorbidity is associated with reduced adherence to diabetic treatment and self-management [9] and poorer glycaemic control with increased risk of diabetes complications [10]. Obesity and T2D are also associated with binge-eating disorder (BED). BED is defined by the Diagnostic and Statistical Manual of Mental Disorders, Fifth edition (DSM-5) by frequent, recurrent episodes of binge-eating characterised by eating, in a discrete period of time, an amount of food that is definitely larger than what most people would eat in a similar period of time under similar circumstances, causing marked distress, occurring at least once a week for 3 months, and without any compensatory behaviour [11]. A literature review of studies in four Latin American countries reported a prevalence of BED of 16–51.6% among obese people attending weight loss programs [12]; whilst World Health Organisation Mental Health Surveys found that people with BED were 3–6 times more likely to be obese compared to those without [13]. Studies have reported a point prevalence of up to 25% in T2D, versus 1.4% in the general population [14] and having BED increases the risk of incident T2D by 6.5-fold [13].

Recently, evidence has emerged demonstrating benefits of GLP-1 receptor agonists in other medical conditions e.g. polycystic ovarian syndrome [15] and mental disorders. For example, liraglutide, a GLP-1 receptor agonist, improved cognitive function in *n* = 19 patients with major depressive disorder (MDD) or bipolar disorder in a pilot randomised controlled trial (RCT) [16], whilst a systematic review and meta-analysis reported a pooled reduction in depressive symptoms with GLP-1 receptor agonists compared to other antidiabetic therapies [17].

GLP-1 receptor agonists have known satiety-promoting effects due to central action of the neuropeptide GLP-1 [1], with reduced activation of appetite- and reward-related brain areas in response to food [18]; thus, they were hypothesised as a treatment for BED [19, 20]. An open-label RCT in *n* = 44 patients with obesity and subclinical binge-eating found a greater reduction in binge-eating at 12 weeks in liraglutide versus control (diet and exercise) groups [20]. Another open-label RCT observed that dulaglutide (another GLP-1 receptor agonist) led to greater reduction of binge-eating behaviour compared to gliclazide in people with T2D and BED [21].

## Aims

The aim of this study was to describe experiences of self-administering GLP-1 receptor agonists and its perceived impact on mental health in patients with obesity and/or T2D.

### Methods

#### Design

This was a qualitative study using individual semi-structured interviews. The Consolidated Criteria for Reporting Qualitative Studies (COREQ) checklist were followed, which is found in the supplementary material (p6).

## Setting

The study was carried out in two settings. One was the public funded National Health Service tier 3 weight management programme in an inner-city teaching hospital in south-east London, United Kingdom (UK*). In the UK*,* weight management services are organised in a four-tier model: community based*,* primary care*,* medical therapies and surgical interventions.* The tier 3 programme is delivered by a multidisciplinary team including specialist weight management dietitians, physiotherapists, psychologists and obesity physicians over one year [22]. Participants took part in a programme using a gradual calorie reduction or a very low-calorie diet. Participants with obesity and pre-diabetes were prescribed subcutaneous liraglutide titrated from 0.6 mg to 3 mg once daily over two months [22]. In the UK, liraglutide is approved for obesity treatment in patients with BMI > 35 kg/m^2^ and non-diabetic hyperglycaemia (glycated haemoglobin (HbA1c) 42–47 mmol/mol) and at high risk of cardiovascular disease.

The second setting was in two community diabetes services in south-east London, UK, which consist of consultant diabetologists, primary care physicians with a special interest in diabetes, diabetes specialist nurses and dieticians. Patients with T2D were prescribed either oral semaglutide (titrated up from 3 mg to 14 mg once daily), subcutaneous semaglutide (titrated from 0.25 mg to 1 mg once weekly), or subcutaneous liraglutide (titrated from 0.6 mg to 1.8 mg once daily) over one month. In the UK, GLP-1 receptor agonists are approved as third line treatment for patients with T2D with a BMI of ≥ 35 kg/m^2^ and specific psychological or other medical problems associated with obesity; or with a BMI < 35 kg/m^2^ if insulin therapy would have significant occupational implications, or weight loss would benefit other obesity-related comorbidities [23].

## Participant selection and recruitment

Potential participants were identified from these services and invited to participate if they were being newly commenced on these medications during the study period (18/09/2022–04/05/2023). Eligibility criteria were: being prescribed a GLP-1 receptor agonist for management of either for obesity or T2D; being at least 18 years of age; capacity to consent; able to converse and read in fluent English.

## Participants

Twenty-five patients consented to be contacted by a member of the research team, of which 13 consented to take part in the study prior to initiation of the GLP-1 receptor agonist and completed baseline questionnaires. Of these, four were lost to follow-up and nine participants took part in the interview 12–16 weeks after treatment.

### Participant characteristics

Demographic data including age, ethnicity, and gender were collected at baseline. Weight (kg) and BMI (kg/m^2^) were recorded prior to initiation and after 12–16 weeks of treatment with GLP-1 receptor agonist; HbA1c levels (mmol/mol) were also recorded for participants with T2D. Mental health status was collected at baseline and 12–16 weeks of treatment. Three screening questionnaires, the Patient Health Questionnaire-9 (PHQ-9) [24], Generalised Anxiety Disorder-7 (GAD-7) [25] assessment and Eating Disorder Examination Questionnaire (EDE-Q) [26] were used for symptoms of depression, anxiety and eating disorders respectively. The PHQ-9 asks 9 questions on depressive symptoms mapped onto DSM-5 criteria for major depressive disorder (MDD) [11]; each item scores from 0 to 3, giving a final score of 0 to 27. Higher scores reflect greater severity and a cut off of 10 represents caseness for MDD [27]. The GAD-7 is a screening tool and severity measure for generalised anxiety disorder. Each item is scored from 0 to 3, giving a range of scores of 0 to 21; higher scores indicate a greater severity and a threshold of 10 has high sensitivity and specificity for diagnosis of generalised anxiety disorder [25]. The EDE-Q is a 28-item questionnaire adapted from the clinician-based interview Eating Disorder Examination, designed to assess the range and severity of disordered eating using four subscales (Restraint, Eating Concern, Shape Concern and Weight Concern) and a global score [26].

## Data collection

Semi-structured individual interviews were conducted with participants 12–16 weeks after starting GLP-1 receptor agonist, to explore experiences of the impact on mental health. The semi-structured nature of the interviews allowed for open-ended questions to be asked, designed to elicit discussion, which were guided by an interview schedule, to ensure the interviews were systematic and comprehensive [28]. Interview topics included: overall experience of taking the medication; side effects experienced; change in physical health; mental health; general wellbeing; eating behaviours and binge-eating.

The interview schedule was developed through discussion with healthcare professionals including a clinician working in a tier 3 obesity service, a diabetes physician, and a psychiatrist specialising in diabetes and mental health, as well as a member of the public with lived experience of taking a GLP-1 receptor agonist (see supplementary material p3). A pilot interview to gauge the comprehensibility and flow of the interview questions was conducted prior to the commencement of formal interviews with this member of the public.

Interviews were conducted by one researcher virtually using the video-conferencing software Microsoft Teams. With informed consent, interviews were audio-recorded and automatically transcribed using Microsoft Teams on encrypted devices, with corrections to the transcription made. Identifiable information was removed from transcripts.

### Data analysis

Interview transcripts were analysed using reflexive thematic analysis to identify, analyse and report patterns within the data. Two authors carried out the analysis, following Braun and Clarke’s guide involving the steps of: 1 familiarisation of data; 2 generation of codes; 3 combining codes into themes; 4 reviewing themes; 5 determining significance of themes; 6 reporting of findings [29].

A combined deductive/inductive approach was used to explore the data according to the research aims, in addition to identifying further themes suggested from the data itself. An audit trail was kept to establish the confirmability of our findings. *Clinical data including changes in physical health measures (weight*,* BMI and HbA1c)*,* and their mental health symptomatology screening*,* were used to describe the study sample and give context to the experiences of the individual participants.*

### Study team and theoretical perspective

The study was conducted by a cross-disciplinary team, with expertise in psychology (MB), diabetes (KI, PSG, AP) and psychiatry (KI, AP). AP is a clinical academic doctor, specialising in endocrinology, and received training in qualitative research; MB is a chartered psychologist with recognised expertise in qualitative methods; KI is a senior clinical academic in liaison psychiatry specialising in diabetes and obesity; PSG is a consultant diabetologist and obesity physician.

## Results

### Participant characteristics

Individual participant characteristics are described in Table 1. Of the nine participants, the majority were female and of white ethnicity. The mean (standard deviation) age of participants was 57 (8) years. Five participants were prescribed GLP-1 receptor agonists for obesity management; four for T2D. The participants on average lost 5.4 (5) % of their body weight from baseline to follow-up. BMI reduced by a mean of 2.7 (3) kg/m^2^. The four participants treated for T2D had a mean reduction in HbA1c of 17 (6) mmol/mol. Individual participant change in PHQ-9, GAD-7 and EDE-Q scores are shown in Table 1.

**Table 1:** Participant characteristics

Patient identifier	Age	Sex	Ethnicity	*Pre-existing mental disorder*	Indication for GLP-1-RA	GLP-1-RA drug name/ max dose achieved^1^/route	BMI stage 1 (kg/m^2^)	BMI stage 2 (kg/m^2^)	HbA1c stage 1 (mmol/mol)	HbA1c stage 2 (mmol/mol)	*PHQ9 stage 1*	*PHQ9 stage 2*	*GAD stage 1*	*GAD stage 2*	*EDE-Q stage 1*	*EDE-Q stage 2*
**1**	64	M	White	N/A	Obesity	Liraglutide 3 mg daily SC	37.1	34.9	N/A	N/A	7	7	5	4	2.3	1.1
**2**	43	M	White	BPD, BED, depression, anxiety, agoraphobia	Obesity	Liraglutide 3 mg daily SC	66.0	58.5	N/A	N/A	23	6	10	3	5.8	2.3
**3**	56	F	Black	N/A	Obesity	Liraglutide 3 mg daily SC	64.5	57.5	N/A	N/A	14	22	8	18	2.8	3.9
**4**	57	F	White	Depression	Obesity	Liraglutide 3 mg daily SC	46.1	48.9	N/A	N/A	16	4	3	0	2.7	1.5
**5**	74	F	White	N/A	Obesity	Liraglutide 3 mg daily SC	56.3	52.6	N/A	N/A	7	6	0	0	2.0	1.3
**6**	56	F	Black	N/A	Diabetes	Liraglutide 1.8 mg daily SC	37.2	33.5	84	62	12	4	10	3	0.6	1.4
**7**	53	M	Mixed race	ASD, depression	Diabetes	Semaglutide 7 mg daily oral	34.5	33.7	90	68	15	20	14	15	4.7	3.0
**8**	59	F	White	Fibromyalgia, depression	Diabetes	Semaglutide 0.5 mg weekly SC	32.5	31.3	84	68	18	11	14	4	2.6	1.2
**9**	53	F	White	N/A	Diabetes	Liraglutide 1.2 mg daily SC	31.0	29.6	67	60	6	10	2	0	3.0	0.2
1. Max dose achieved refers to the maximum dose of GLP-1 receptor agonist reached during titration at stage 2. 2. Stage 1 refers to baseline data prior to initiation of GLP-1 receptor agonist; stage 2 refers to the time of the interview at 12–16 weeks follow-up. Abbreviations: ASD: autism spectrum disorder, BED: binge eating disorder; BMI: body mass index, BPD: borderline personality disorder, EDE-Q: Eating Disorder Examination Questionnaire, GAD: Generalised Anxiety Disorder questionnaire, GLP-1-RA: glucagon-like peptide-1 receptor agonist, PHQ-9: patient health questionnaire-9, SC: Subcutaneous																

### Themes

**Fig. 1:**
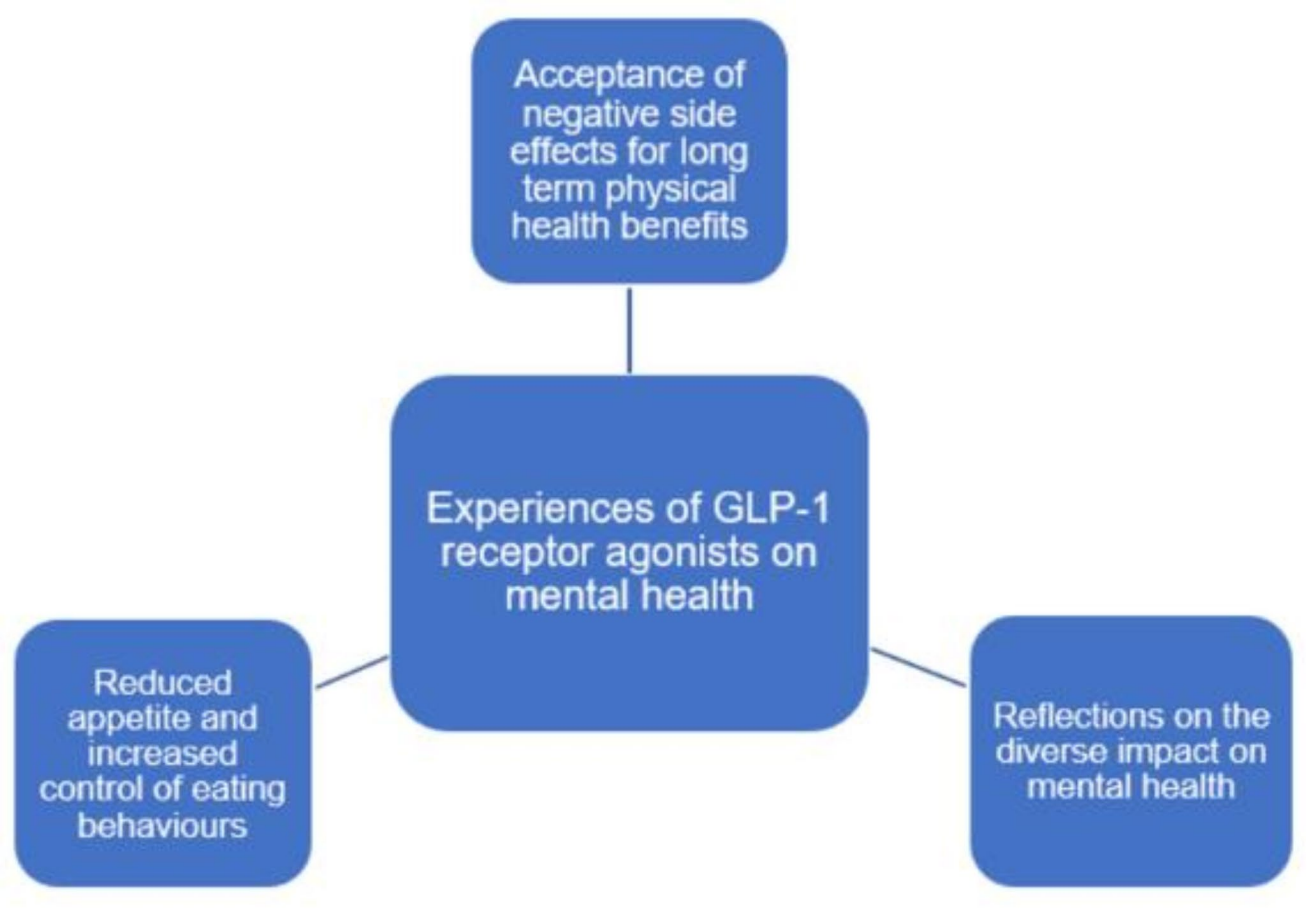
Generated themes from thematic analysis

Analysis resulted in the generation of three overarching themes related to experiences of patients taking GLP-1 receptor agonists (Fig. 1).

Quotations from the participants are presented using best exemplars to illustrate the themes.

We included the gender and indication for GLP-1 receptor agonist for the participant in the descriptor of each quote.

### Theme 1: Acceptance of negative side effects for long term physical health benefits

Most participants described significant weight loss following treatment with GLP-1 receptor agonists. Concurrently, many reported a change in their appearance, including in face shape and clothing size.

“The weight is slowly coming off…two months after taking it I’d lost 10 kilos…a year ago, I bought an emergency suit because nothing fitted…today it hangs like a sack.” (P1, male, obesity).

“My trousers have started falling down. I have to keep adjusting my belt…One day my face ID on my phone stopped working…my face ID isn’t recognising me.” (P2, male, obesity).

Participants with T2D described improved blood glucose control and reduction in HbA1c values at medical appointments. One participant with obesity reversed their prediabetes.

“I went from [a HbA1c of] 47 to 35. So I’m no longer prediabetic.” (P1, male, obesity).

In some cases, this allowed reduction in dose of other medications such as insulin which they had often been taking for many years. This was viewed as very positive as it indicated better diabetes control.

“I came off the Novorapid [short-acting insulin]…and there’s a kind of instant effect of the weight coming down.” (P9, female, diabetes).

Several participants reflected on improvements in a range of physical health issues including breathlessness, back pain, frequent urination and urine infections, sleep, and skin problems.

“I ran for a bus…a year ago I couldn’t have walked to the end of my street without needing a break…I’m certainly feeling healthy.” (P1, male, obesity).

“The breathlessness - it’s just gone…Whereas before just going to the bathroom or kitchen…I’d be puffing and panting.” (P2, male, obesity).

Many participants experienced gastrointestinal side effects including abdominal pain, diarrhoea, nausea and vomiting, acid reflux and constipation, which varied in severity.

“All of a sudden I started vomiting.” (P3, female, obesity).

“It’s horrendous…Every time I think of food, I feel sick to my stomach…It was unbearable at first.” (P8, female, diabetes).

Negative side effects typically improved over time, with many participants having no or minimal side effects at 12–16 weeks. Side effects often temporarily worsened when the dose was uptitrated.

“Every time I went up a little bit I would feel nauseous for like a week and then it would settle down.” (P9, female, diabetes).

Universally, participants were willing to tolerate these side effects for improvements in physical health including their weight.

“I’m quite happy to put up with it to keep this weight going down.” (P5, female, obesity).

Some participants described initial concerns about self-injecting and their ability to tolerate the needles, particularly those who were not self-administering insulin already. In most cases they found the injections to not be a problem upon initiation.

“One of my original concerns was would I be able to tolerate injections every day.” (P1, male, obesity).

“They’re the tiniest little things…You don’t feel them.” (P4, female, obesity).

“I’m used to it… It’s basically the same [as insulin].” (P9, female, diabetes).

Several participants expressed a desire for increased availability of the medication to other people, and were aware that the funding was only for a limited period.

“I really appreciate the program. And I hope it’s something that is more widely available soon. It can make a big difference.” (P1, male, obesity).

“I’m their poster boy now. There’s no doubt that I’ll be paying for it after those two years…I wish everyone that needed it could access it.” (P2, male, obesity).

Some participants favourably compared GLP-1 receptor agonists with other treatment options such as bypass surgery for obesity.

“I think it really helped me. I helps you lose weight faster…I could say maybe it will be better than the bypass surgery.” (P3, female, obesity).

### Theme 2: reflections on the diverse impact on mental health

The majority of participants noticed an improvement in their general wellbeing and increased hopefulness after having struggled with their condition for many years.

“It’s had a positive effect…I feel more hopeful.” (P9, female, diabetes).

Several patients reported improvements in mood, including patients who felt depressed prior to starting the medication.

“My general mood is so much better.” (P2, male, obesity).

This was reflected by a large reduction in their PHQ-9 score of 17 points and accompanied by significant improvements in physical health: P2 lost over 11% of their body weight.

One participant described reduced hopelessness and suicidal ideation.

“I think I’m less depressed…I don’t have such kind of low mood…I was having some suicidal ideation…thoughts of ‘what’s the point’, that kind of pointless, hopeless type of thinking. And I don’t have that anymore.” (P9, female, diabetes).

Interestingly, however, this participant’s PHQ-9 score increased from 6 to 10: when individual items were examined, mood stayed the same with the change in score secondary to increased fatigue and change in appetite. They had a reduction in HbA1c from 67 to 60 mmol/mol. Another participant also described reduced psychological distress but had an increase in PHQ-9 score (P3).

Other participants discussed reduced anxiety levels secondary to better control of blood glucose and/or food intake.

“Being able to eat and not really having to worry about it because it’s [the medication] doing it for me. It’s taking all the worry out of the food going in.” (P8, female, diabetes).

This participant had a corresponding large reduction in their GAD-7 score of 10 points and a drop in HbA1c from 84 to 68 mmol/mol.

One participant who suffered from borderline personality disorder (BPD) experienced a significant improvement in their mental health such that they were discharged from their psychiatry team.

“My community mental health team signed me off… I had been due to start DBT [dialectical behaviour therapy], and even before my assessment appointment, I was kinda like I’m not sure I actually need this anymore.” (P2, male, obesity).

This was confirmed by their reduction in PHQ-9 score of 17 points and of their GAD-7 score by 7 points, with an associated weight loss of over 11%. Importantly, they noted that these mental health improvements had not occurred when they had achieved this level of weight loss in the past through other means e.g. strict dieting; thus, there appeared to be an additional direct psychological effect of GLP-1 receptor agonists, rather than the weight loss per se.

Several participants reported improvements in self-esteem, self-confidence and body image, which was not always explained by weight loss, as similar changes in confidence had not been experienced in previously successfully weight loss methods.

“[I’m] feeling like I’m a worthy person…and I deserve my place on Earth…My confidence is definitely improved…Again, it wouldn’t have improved at this point when I’ve lost weight before.” (P2, male, obesity).

Some participants described increased resilience, ability to cope and self-control with negative emotions following initiation of a GLP-1 receptor agonist.

“Someone in the group accused us of excluding everyone else and the reality is we didn’t…When I first read that message, it bothered me, but I was able to let go of it really quickly and kind of not let it affect me.” (P2, male, obesity).

One participant with a history of depression experienced a new episode of depression during the study with an associated increase in PHQ-9 of from 15 to 20 points despite an initial sense of increased resilience. Of note, the score at baseline prior to initiation of GLP-1 receptor agonist was already in the moderately-severe depression range.

“It felt when I was initially taking the medication…when I was feeling down… I felt an initial ability to process and cope better and quicker. (P7, male, diabetes)

Improvements in social and interpersonal relationships were described, often related to an improvement in the participants’ overall mood and social confidence.

“I’ve got a great relationship with my children…But I would say that’s improved actually…We’re having more fun…[I feel] a little bit more sociable…a little bit more outgoing.” (P9, female, diabetes).

However, some participants reported no subjective change in their mental state, particularly if they had no concerns at baseline.

“I feel exactly the same. I think after being so unwell mentally two and a bit years ago, I think I’d notice.” (P4, female, obesity).

Interestingly, these two participants actually had reductions in their PHQ-9 score. P4’s PHQ-9 score reduced from 16 to 4 i.e. from moderately severe depression to minimal symptoms, and was the only participant to not lose weight during the study, with an increase of 7 kg; P6’s PHQ-9 score reduced from 12 to 4, i.e. from moderate depression to minimal symptoms and despite a large reduction in HbA1c from 84 to 62 mmol/mol.

### Theme 3: reduced appetite and increased control of eating behaviours

The most consistent theme emerging from the interviews was changes in eating behaviours. All participants described reduced appetite due to increased satiety. This led to a significant reduction in portion sizes, as well as long periods without eating, due to lack of hunger.

“I just lost appetite. I don’t even feel hungry in the whole day. Sometimes I forget about eating.” (P3, female, obesity).

“My dinner size has gone down by half.” (P8, female, diabetes).

Some participants described relying on other external cues to eat, or forcing themselves to eat as they did not feel hungry.

“I have to listen to my body for other cues that I need to eat, because I can quite happily go out, have a full active day, and not feel the need to eat at all.” (P1, male, obesity).

Participants were overall extremely positive about the appetite-supressing effect of the medication, which was what they were hoping for.

“I’m really enjoying the appetite suppressant element of it.” (P9, female, diabetes).

In some cases, the reduced appetite was initially attributed to nausea or abdominal pain, though the low appetite persisted once side effects wore off.

“I can feel that I’m eating too much, and then it makes me feel like I want to be sick.” (P6, female, diabetes).

In most cases, the types of foods eaten did not change, and specifically “healthier” foods were not chosen; rather it was quantities that were reduced.

“I’m still eating exactly the same, just less.” (P5, female, obesity).

In a small number of participants, food choices were altered to minimise nausea and reflux, and in some cases this led to less healthy food choices.

“It basically feels like morning sickness…you just want to eat bland food.” (P9, female, diabetes).

“the quality of my food went down…much more chocolate, more carb-based things.” (P9, female, diabetes).

The majority of participants reported reduced cravings and snacking in between meals. This was often experienced as a psychological effect rather than simply due to reduced hunger.

“It’s eliminated cravings.” (P1, male, obesity)

“I don’t snack as much now….Crisps, pringles, that would be a craving…I don’t even touch them now.” (P8, female, diabetes).

Participants who previously engaged in comfort or stress eating experienced reductions in these behaviours. They discussed having increased mindfulness around eating and being more intentional when making decisions about food choices.

“There’s less kind of temptation to eat in the evening…whereas before I could be tempted to pick and watch a bit of telly and eat some food.” (P9, female, diabetes).

“The mindfulness of like ‘I don’t really want that, you know, what are you getting it for?’” (P4, female, obesity).

These reductions in cravings and in emotional eating were associated with a decrease in scores in the EDE-Q scores, e.g. P9 and P4 had reductions of 2.8 and 1.3 points respectively. Although this was usually accompanied by weight loss, P4 was the only participant to gain weight (7 kg) during the study. However, this participant had a slower dose titration due to their heart failure, so took longer to reach the maximum dose; as a result, they stopped taking the medication prior to the follow-up interview as they felt it was not effective. Interestingly, they described reducing cravings whilst on the medication which then returned after stopping.

Many participants felt they had increased control of their eating behaviours and could make better choices regarding the food they ate and the quantities they consumed.

“I felt I had more control over my eating…I felt I was better able to make choices.” (P7, male, diabetes).

“It’s taken my 74 years to learn what portion control can really look like.” (P5, female, obesity).

Two participants reported *obsession* with food prior to starting a GLP-1 receptor agonist, one of whom was diagnosed with BED. Both described previously struggling with constant ruminations about food, even just after finishing a meal, which improved dramatically with treatment, and P2’s BED remitted. They reported reduced drive to eat, in addition to less physical hunger.

“It’s removed the ‘I am starving’ half an hour after I’ve eaten, that I’ve had all my life…I’ve never not felt hungry in a way…I could sit down and eat a meal and then by the time you’re sort of finished talking I knew I was hungry again…I can think right back to childhood, I’ve always felt hungry and just thought it’s cause I’m a greedy pig.” (P5, female, obesity).

“I’ve lived with binge-eating disorder for like 25 years…Literally overnight, when I started taking it, it was like a switch had been turned off in my brain and all my thoughts about food vanished…So the binge-eating has gone completely. I’ve not had a single binge episode in all that time.” (P2, male, obesity).

This was reflected in their EDE-Q scores which reduced by 3.5 and 0.7 points for P2 and P5 respectively.

## Discussion

We examined the experiences of patients with obesity or T2D attending medical weight management or community diabetes service respectively, who were prescribed a GLP-1 receptor agonist, with respect to changes in their mental health including eating behaviours.

We observed three main themes. The first pertained to physical health effects, with participants reporting an overall positive experience of taking the medication, finding that the improvements in weight and blood glucose control outweighed the gastrointestinal side effects; some found taking GLP-1 receptor agonists to be transformational. The second demonstrated a range of mental health impacts of the medication, with some participants experiencing improvements in mood, anxiety, confidence, self-esteem and interpersonal relations, whereas others felt no different in these regards. The third, and most ubiquitous theme, was a change in eating behaviours, both physical, with reduced appetite and increased satiety, as well as psychological, with increased control of eating, and reduction in cravings, comfort eating and food obsession.

*Comparing the obesity and diabetes sub-cohorts*,* the overall experience appeared to be more positive for those being treated for obesity*,* who were often delighted with the effect of the treatment and wished for greater availability of the medication. This may be because the physical health benefits (i.e. weight loss) in this group were more obvious than the improved blood glucose levels seen in the diabetes group. In addition*,* the majority of participants felt the most beneficial aspect of the medication was the increased control of eating behaviours*,* which was of more importance to the obesity cohort in view of the primary aim of the medication being weight loss. Interestingly*,* participants did not report specifically on quality-of-life improvements*,* including for example change in sexual function which has been demonstrated previously with GLP-1 receptor agonists* [30].

The strengths of this study include, to our knowledge, that this is the first study to qualitatively describe the experiences of patients prescribed GLP-1 receptor agonists with respect to mental health and eating behaviours. Only one qualitative study has been conducted to date investigating patients taking GLP-1 receptor agonists [31], which focussed on reasons for adherence to the medication. Second, our sample was diverse in terms of ethnicity, gender and socioeconomic status and for being set in the public sector and in the inner city. Third, the study is unique in also using validated questionnaires for mental disorders common in obesity and T2D to standardise the baseline mental health characteristics of the participants and attempt to substantiate and further interpret the subjective experience of change in mental health with change in scores. Fourth, involving the public in the development of the topic guide ensured the research was more relevant to the needs and preferences of patients affected by obesity and T2D. Finally, collecting biomedical as well as qualitative data allowed interpretation of subjective experiences in the context of changes in physical health parameters.

There are several important limitations. First, the small sample size was secondary to: (a) a national shortage of GLP-1 receptor agonists during the study period [32] with pharmacy availability issues; (b) a paucity of computer literacy in the patient population, meaning several potential participants were unable to use MS Teams for the interviews; and (c) a slow return to usual care post-Covid-19 pandemic. *Thus*,* this could be considered an exploratory study*,* with further data required for definitive conclusions to be drawn*. Second, we did not assess adherence to medication in the sample; for example, P5 only took liraglutide for 1 month due to logistical issues, whilst P4 stopped just prior to the follow-up appointment due to perceived lack of efficacy. Some participants may have been reluctant to report poor adherence to maintain social desirability. Finally, duration of follow-up was only 12–16 weeks; a longer follow-up period would be useful to identify long-term effects of GLP-1 receptor agonists on mental health and effects of newer, perhaps more efficacious GLP-1 receptor agonists at higher doses which have since been approved e.g. subcutaneous semaglutide (up to a dose of 2.4 mg weekly), or dual gastric inhibitory polypeptide (GIP)/GLP-1 receptor agonists e.g. tirzepatide, approved for T2D and undergoing appraisal currently by the National Institute for Health and Care Excellence (NICE) for obesity.

Our findings suggest that most experienced improvement in mental health but not necessarily secondary to weight loss. *This may in part be due to a positive perception of the relative ease of weight loss with the medication.* However, it additionally raises the possibility of a direct brain effect of GLP-1 receptor agonists that could target mental disorders common in obesity and T2D. Participants were able to distinguish between a physical effect e.g. reduced hunger, from a psychological effect, namely change in cognitions (reduced thinking about food) and affect (reduced anxiety). Landmark preclinical studies using animal knockouts have demonstrated that GLP-1 receptors in the brain are capable of controlling food intake and body weight, in addition to the hypophagic effects of endogenous GLP‐1 via peripheral GLP‐1 receptors [33]. Data suggest the requirement of GLP‐1 receptors in both the vagal afferents as well as the brain for the full effects of GLP‐1 receptor agonists on energy balance, with the hypothalamus being the primary downstream target of peripherally administered GLP‐1 receptor agonists. An RCT in obese patients found that GLP-1 receptor activation by the agonist exenatide reduced brain responses on functional MRI to food cues in appetite- and reward-related brain areas including the insula and amygdala, correlating with reductions in food intake [18]. Furthermore, a systematic review demonstrated beneficial effects of GLP-1 in reward system-related disorders pertaining to both drugs of abuse and palatable foods [34]. These findings are in keeping with pilot clinical studies showing a beneficial effect of GLP-1 receptor agonists on binge-eating [20, 21] and confirms the need for larger clinical trials of this class of drugs in BED.

Despite not being a primary aim of the study, by collecting participant characteristics we saw clinically significant improvements in physical health (weight and HbA1c), of similar magnitude to that seen in trials of GLP-1 receptor agonists in obesity [35] and T2D and overweight [36, 37]. This suggests our sample was representative of those who could benefit from GLP-1 receptor agonists.

Although this was an unselected sample of participants with respect to mental illness, nearly all participants had clinically significant depressive symptoms and anxiety. This is in keeping with the known association between obesity and T2D and psychiatric illness. Although the purpose of the study was not to conduct quantitative analyses, questionnaire data showed a trend towards reduction in PHQ-9, GAD-7 and EDE-Q scores (Table 1), which were clinically significant given that change scores of 5, 4 and 0.57 are considered the minimal clinically important differences in PHQ-9, GAD-7 and total EDE-Q scores respectively [38–40]. We would therefore recommend powered quantitative studies investigating changes in psychiatric symptoms pre- and post-treatment with GLP-1 receptor agonists. In particular, studies investigating whether patients would tolerate negative side effects for mental health benefits are warranted, as this was not specifically found in our study, though universally participants accepted the side effects for physical health benefits.

### Implications

This study adds to growing evidence that GLP-1 receptor agonists have a positive effect on mental health, and thus could be a particularly good treatment option for patients with obesity and/or T2D with comorbid psychiatric illness. It remains unclear whether these drugs are beneficial in people with anxiety or depression who do not also have obesity or T2D, which also warrants further investigation. Our study importantly suggests that the beneficial effects on mental health were not entirely explained by weight loss; thus, there may be a direct brain effect leading to improved mood. Our study concurs with previous data regarding the efficacy of GLP-1 receptor agonists in binge-eating disorder, as demonstrated by the remission of the patient with BED at baseline. We therefore hypothesise that this class of medication could be repurposed for BED, and larger scale clinical trials should be planned to investigate this.

## Conclusion

This study highlights the wide-ranging impacts of GLP-1 receptor agonists on mental health outcomes. Findings have highlighted the potentially beneficial effect on mental health and the potential to be repurposed for BED.

## Electronic supplementary material

Below is the link to the electronic supplementary material.

S1 file: Interview schedule for semi-structured interview. S2 fie: COREQ checklist

## Data Availability

The data are available from the corresponding author upon reasonable request.
