# Supplementary material for: A qualitative study of the mental health outcomes in people being treated for obesity and type 2 diabetes with glucagon-like peptide-1 receptor agonists
Source: Acta Diabetol. 2024 Nov 9;62(5):731–42. doi: 10.1007/s00592-024-02392-0 (PMC12116896; doi:10.1007/s00592-024-02392-0)
Supplement: Supplementary file 1 — S1 file: Interview schedule for semi-structured interview. S2 fie: COREQ checklist [file 592_2024_2392_MOESM1_ESM.docx]

**Supplementary material: A qualitative study of the mental health outcomes in people being treated for obesity and type 2 diabetes with glucagon-like peptide-1 receptor agonists**

**Journal:** Acta Diabetologica

**Authors:** Aureliane Pierret (MBBChir)^1,2^, Madeleine Benton (PhD)^2^, Piya Sen Gupta (PhD)^1^, Khalida Ismail (MRCPsych)^2^

**Affiliations**:

1. St Thomas’ Hospital, Guy’s and St Thomas’ NHS Foundation Trust, Westminster Bridge Road, London, SE1 7EH
2. Department of Psychological Medicine, King’s College London, 16 De Crespigny Park, London, SE5 8AB

**Corresponding author:**

Aureliane Pierret

[a.pierret@nhs.net](mailto:a.pierret@nhs.net)

Contents

[**Interview schedule for semi-structured interview** 3](#_Toc168575424)

[**COREQ (COnsolidated criteria for REporting Qualitative research) Checklist** 6](#_Toc168575425)

# **Interview schedule for semi-structured interview**

Title of Research Project: GLP1 receptor agonists and mental health

IRAS ID: 314639

**Overview, aims and consent**

1.Introduction

Hello, my name is Aureliane. I’m one of the researchers working on this project about GLP1 receptor agonists, the type of medication you have recently started taking.

2. Aims of the study

The group conducting the study is part of the psychiatry department at KCL, and they focus specifically on the overlap between psychology, diabetes and obesity.

Our aims in this study are looking at the effect of GLP1 receptor agonists on mental health, wellbeing, and eating behaviours.

The interview will involve a number of questions about your experience taking this medication, and any effect it has had on you personally. This part of the interview should take about 30 minutes.

It will be audio recorded so that I can go back and listen to the interview to make sure I don’t forget anything. The interviews will be anonymous so if we publish a paper about what people have said, no-one will know that it was you.

Thank you for providing me with the consent form. Before we get started do you have any more questions about the study?

**Body**

**Overall experience**

*I’m going to start by asking you some questions about your overall experience of taking this medication.*

1. What were your thoughts about the medication before you started taking it?

2. How have your thoughts about it changed since you started taking it?

**Effect on physical and mental health**

*I’m now going to ask you some questions about how you feel the medication has affected your physical and mental health, and general wellbeing.*

3. How do you think your physical health has changed since starting this medication?

*Prompts: for example, reduced weight, increased fitness, better blood sugar levels*

4. How do you think your mental health has changed since starting this medication?

*Prompts: for example, improved mood, reduced anxiety*

5. Are there any other changes you have noticed, aside from your physical and mental health?

*Prompts: for example, changes in social life, confidence in themselves*

6. When did you start noticing the main changes in your physical health after starting the medication?

*if they noticed a change in physical health

*Prompts: days, weeks, months?*

7. When did you start noticing the main changes in your mental health after starting the medication?

*if they noticed a change in mental health

*Prompts: days, weeks, months?*

8. These changes you’ve noticed, do you think they are still continuing now?

*specific to interviewee – tailor this question to their responses above

*Prompts: For example, is your appetite still as low as when you started? have you managed to maintain the weight loss?*

9. If not, at what point did you feel the effect(s) starting to wear off?

*Prompts: e.g. at what point did your weight start to stabilise or increase again?*

10. How do you think X has impacted on your wellbeing in general?

*Prompts: for example, are there any differences in how you feel on an average day now compared to before? how do you feel in yourself compared to before?*

**GLP1 and eating habits**

*Now I’m going to ask you some questions about your eating habits and how these might have been affected by taking GLP1 agonists.*

10. How would you describe your eating habits before starting on GLP1?

*Prompts: did you eat regular meals? did you snack a lot? would you say you had a healthy diet? would you say you ate a lot of food or not very much food?*

11. How have your eating habits changed since starting GLP1?

*Prompts: e.g. less appetite, smaller portions, less snacking, healthier choices*

12. Prior to starting with GLP1, can you tell me what your relationship with food was like?

*Prompts: e.g. was it only to fuel yourself for survive; used as a reward; something you had control over or not?*

13. How has your relationship with food changed since starting GLP1?

*Prompts: E.g. no longer as rewarding, more control*

**GLP1 and binge eating**

*Now I’d like to ask you a few questions about binge eating specifically. The definition of binge eating is “a period of time where a person loses control over their eating behaviour, and eats considerably more than usual and feels unable to stop eating or limit the amount of food eaten”*

14. Would you say you’ve ever experienced binge eating in the past?

15. *If yes – Can you tell me how starting GLP1 has impacted on those binge eating experiences?

**Additional information**

Is there anything else that you would like to share about your experiences?

**Summary**

Thank you so much for taking part in this interview. It has been really useful to hear about your experiences taking this medication.

# **COREQ (COnsolidated criteria for REporting Qualitative research) Checklist**

A checklist of items that should be included in reports of qualitative research. You must report the page number in your manuscript where you consider each of the items listed in this checklist. If you have not included this information, either revise your manuscript accordingly before submitting or note N/A.

| **Topic** | **Item No.** | **Guide Questions/Description** | **Reported on Page No.** |
| --- | --- | --- | --- |
| **Domain 1: Research team and reflexivity** |  |  |  |
| *Personal characteristics* |  |  |  |
| Interviewer/facilitator | 1 | Which author/s conducted the interview or focus group? | 11 |
| Credentials | 2 | What were the researcher’s credentials? E.g. PhD, MD | 1 |
| Occupation | 3 | What was their occupation at the time of the study? | 13 |
| Gender | 4 | Was the researcher male or female? | N/A |
| Experience and training | 5 | What experience or training did the researcher have? | 13 |
| *Relationship with participants* |  |  |  |
| Relationship established | 6 | Was a relationship established prior to study commencement? | Supporting information |
| Participant knowledge of the interviewer | 7 | What did the participants know about the researcher? e.g. personal goals, reasons for doing the research | Supporting information |
| Interviewer characteristics | 8 | What characteristics were reported about the inter viewer/facilitator? e.g. Bias, assumptions, reasons and interests in the research topic | Supporting information |
| **Domain 2: Study design** |  |  |  |
| *Theoretical framework* |  |  |  |
| Methodological orientation and Theory | 9 | What methodological orientation was stated to underpin the study? e.g.  grounded theory, discourse analysis, ethnography, phenomenology, content analysis | 12 |
| *Participant selection* |  |  |  |
| Sampling | 10 | How were participants selected? e.g. purposive, convenience, consecutive, snowball | 10 |
| Method of approach | 11 | How were participants approached? e.g. face-to-face, telephone, mail, email | 10 |
| Sample size | 12 | How many participants were in the study? | 10 |
| Non-participation | 13 | How many people refused to participate or dropped out? Reasons? | 10 |
| *Setting* |  |  |  |
| Setting of data collection | 14 | Where was the data collected? e.g. home, clinic, workplace | 12 |
| Presence of nonparticipants | 15 | Was anyone else present besides the participants and researchers? | 12 |
| Description of sample | 16 | What are the important characteristics of the sample? e.g. demographic data, date | 10, 14 |
| *Data collection* |  |  |  |
| Interview guide | 17 | Were questions, prompts, guides provided by the authors? Was it pilot tested? | 11, 12 |
| Repeat interviews | 18 | Were repeat inter views carried out? If yes, how many? | 10-12 |
| Audio/visual recording | 19 | Did the research use audio or visual recording to collect the data? | 12 |
| Field notes | 20 | Were field notes made during and/or after the inter view or focus group? | 12 |
| Duration | 21 | What was the duration of the inter views or focus group? | N/A |
| Data saturation | 22 | Was data saturation discussed? | N/A |
| Transcripts returned | 23 | Were transcripts returned to participants for comment and/or correction? | N/A |
| **Domain 3: analysis and findings** |  |  |  |
| *Data analysis* |  |  |  |
| Number of data coders | 24 | How many data coders coded the data? | 12 |
| Description of the coding tree | 25 | Did authors provide a description of the coding tree? | 12 |
| Derivation of themes | 26 | Were themes identified in advance or derived from the data? | 12 |
| Software | 27 | What software, if applicable, was used to manage the data? | N/A |
| Participant checking | 28 | Did participants provide feedback on the findings? | N/A |
| *Reporting* |  |  |  |
| Quotations presented | 29 | Were participant quotations presented to illustrate the themes/findings?  Was each quotation identified? e.g. participant number | 17 |
| Data and findings consistent | 30 | Was there consistency between the data presented and the findings? | 17 |
| Clarity of major themes | 31 | Were major themes clearly presented in the findings? | 17 |
| Clarity of minor themes | 32 | Is there a description of diverse cases or discussion of minor themes? | 24 |

Developed from: Tong A, Sainsbury P, Craig J. Consolidated criteria for reporting qualitative research (COREQ): a 32-item checklist for interviews and focus groups. *International Journal for Quality in Health Care*. 2007. Volume 19, Number 6: pp. 349 – 357

**Once you have completed this checklist, please save a copy and upload it as part of your submission. DO NOT** **include this checklist as part of the main manuscript document. It must be uploaded as a separate file.**
